# Factors determining the persistence or recurrence of well-differentiated thyroid cancer treated by thyroidectomy and/or radioiodine in the Boston, Massachusetts area: A retrospective chart review

**DOI:** 10.1186/1756-6614-4-9

**Published:** 2011-04-15

**Authors:** Angela M Leung, Shalini Dave, Stephanie L Lee, Francis X Campion, Jeffrey R Garber, Elizabeth N Pearce

**Affiliations:** 1 Section of Endocrinology, Diabetes, and Nutrition; Boston University School of Medicine; Boston, MA; USA; 2 Division of Graduate Medical Sciences; Boston University Medical Center; Boston, MA: USA; 3 Endocrinology; Harvard Vanguard Medical Associates; Boston, MA; USA; 4 Internal Medicine; Harvard Vanguard Medical Associates; Boston, MA; USA; 5 Division of Endocrinology, Diabetes, and Metabolism; Department of Medicine; Beth Israel Deaconess Medical Center; Boston, MA; USA

## Abstract

**Objective:**

To assess predictors of well-differentiated thyroid cancer (WDTC) persistence/recurrence.

**Design:**

This was a retrospective chart review of thyroid carcinoma patients seen 1979-2007 in a Boston, Massachusetts-area multispecialty group. Of 1,025 patients, 431 met eligibility criteria. Cox proportional hazards models were used to assess predictors (gender, age, ethnicity, tumor size, surgical histology) of WDTC persistence/recurrence (elevated thyroglobulin levels with negative thyroglobulin-antibodies; or positive imaging). Local extension of disease and lymph node involvement could not be assessed.

**Results:**

Mean age at initial surgery (n = 431, 74% women, 79% Caucasian) was 45.8 ± 13.5(SD) years. Mean tumor (papillary, 91%; follicular, 5%; Hurthle cell, 2%; ≥1 type, 2%) size was 2.5 ± 1.6(SD) cm. Most tumors were unifocal (57%) and ≥1 cm (89%). Over 2,600 person-years of follow-up, persistence/recurrence occurred in 52 patients (12%) 4.3 years (median; range 0.2-23.2 years) after surgery. Gender, ethnicity, tumor size, multifocality, and histology were not predictive of persistence/recurrence, while older age was predictive in some models.

**Conclusions:**

In WDTC patients treated by total and near total thyroidectomy and radioiodine and analyzed without consideration of local, locoregional, and distant extent of disease, neither size of tumor nor male gender contribute to disease persistence/recurrence. Age at diagnosis seems to have some positive prognostic value even if only patients older than 21 years at diagnosis are considered. Due to the rare occurrence of follicular (also oxyphilic) histotype, this conclusion refers mainly to patients with papillary thyroid cancer.

## Introduction

Many post-operative scoring systems have been proposed to predict outcomes of well-differentiated thyroid cancer (WDTC). The AMES criteria were developed in the late 1980s and examine age, distant metastasis, tumor extent, and tumor size to predict prognosis in patients with differentiated thyroid cancer [1]. Both the AGES system, which scores age, tumor size, histologic grade, tumor extent, and distant metastasis [2] and the MACIS system, which scores distant metastasis, age, completeness of primary tumor resection, local invasion, and tumor size [3] were established by Hay et al. in the late 1980s and early 1990s as prognostic scoring systems for patients with papillary thyroid cancer. In 1998, the Thyroid Cancer Treatment Cooperative Study Registry Group validated a clinicopathologic staging utilizing age at diagnosis, tumor histology, tumor size, intrathyroidal multifocality, extraglandular invasion, metastases, and tumor differentiation [4]. Finally, the TNM scoring system, which utilizes tumor size, lymph node involvement, and distant metastasis, was developed by the American Joint Committee on Cancer to provide a staging system for all malignancies, including differentiated thyroid cancers [5].

The serum thyroglobulin (Tg) concentration is commonly used as a marker of thyroid tissue persistence/recurrence after near-total thyroidectomy in patients with negative Tg-antibody titers, with recombinant human thyroid stimulating hormone (rhTSH)-stimulated Tg levels >2.5 ng/mL as the optimal level for future recurrence [6]. It may remain elevated for several weeks or months after surgery, and its sensitivity is improved with post-operative remnant radioiodine ablation [7]. Although no single scoring system has been clearly superior, in part due to the differences used in the weighting and combinations of independent predictors, distant metastases, age of the patient, and extent of the tumor seem to be the most consistent predictors of persistent or recurrent disease [8].

Several retrospective analyses have assessed outcomes of low-risk thyroid cancer using these and other factors. In what eventually became the Ohio State University staging system for papillary or follicular thyroid carcinomas, Mazzaferri and Jhiang reported that in 1,355 patients with well-differentiated tumors ≥1.5 cm with or without local or lymph node spread followed over 40 years, near-total thyroidectomy followed by radioiodine ablation and TSH suppression therapy improves outcomes [9]. A review summarizing guidelines by the American Thyroid Association, which have recently been revised [8], the European Thyroid Association, and other recent literature has recommended that total or near-total thyroidectomy followed by remnant ablation is acceptable for those with low-risk papillary thyroid cancer, although surgery alone may be sufficient for some tumors removed incidentally during surgery for benign disease, in the absence of other concerning history [10]. Ongoing research is needed to help clarify the factors which best predict persistence or recurrence of well-differentiated thyroid cancer to guide short-term and long-term management.

The objective of this study was to test the hypothesis that certain patient-associated or tumor-associated factors predict well-differentiated thyroid cancer persistence or recurrence in a Boston, Massachusetts-area cohort of well-differentiated thyroid cancer patients treated with thyroidectomy and radioiodine over a 28-year period.

## Methods

### Study sample

A retrospective chart review was conducted of patients with well-differentiated thyroid cancer evaluated at all sites of a large Boston, Massachusetts-area multispecialty group practice (Harvard Vanguard Medical Associates, HVMA) between 1979 and 2007. Patients with ICD-9 code 193 (thyroid carcinoma) had been identified for a previously-created health quality assurance database at all HVMA sites between January 1979 and May 2007. Of the 1,025 individuals in the preexisting database, 65 were incorrectly coded as having thyroid cancer. Of the remaining 960 potentially eligible patients, 431 (45%) met study criteria and were enrolled in the study.

Inclusion criteria were: 1) at least age 21 at the time of thyroid surgery, 2) history of total or near-total thyroidectomy, 3) diagnosis of papillary, follicular, or Hurthle cell carcinoma by surgical pathology, 3) use of radioiodine ablation post-operatively, and 4) follow-up management within HVMA that included at least one serum thyroglobulin measurement, and/or imaging study by either radioiodine scan, neck ultrasonography, CT, MRI, or FDG-PET. Local extension of disease and lymph node involvement were not included due to the absence of these data in many patients who had their thyroid surgery by non-HVMA providers within the cohort. Exclusion criteria were: 1) medullary thyroid cancer, 2) anaplastic thyroid cancer, 3) incorrectly-coded ICD-9 diagnosis of thyroid cancer, 4) no documentation of thyroid cancer pathology, 5) positive thyroglobulin antibody titers (which make thyroglobulin results uninterpretable) without follow-up imaging, and 6) a positive thyroglobulin level within 6 months of thyroid surgery as the last known status. The date of the thyroid surgery was the entry point for the study. The protocol was approved by the Harvard Vanguard Institutional Review Board.

### Assessment of WDTC persistence or recurrence

Various factors assessing patient demographics and tumor characteristics were grouped to assess their predictive effects on WDTC persistence or recurrence. Gender, race, and age in years at thyroid surgery comprised the patient-associated factors. Tumor size in centimeters and histologic tumor type (e.g. papillary, follicular, Hurthle, or a combination of these) by surgical pathology report comprised the tumor-associated factors. Local extension of disease and lymph node involvement could not be assessed.

Based on the chart review, the persistence or recurrence of well-differentiated thyroid cancer, defined as the primary outcome, was determined at the final time point for which their status was known. For the purposes of this study, thyroid cancer persistence or recurrence were considered identical. Recurrence or persistence of tumor, following total thyroidectomy and radioiodine ablation, was defined as serum thyroglobulin levels greater than 0.5 μg/L (unstimulated) or >2 μg/L (following rhTSH stimulation or thyroid hormone withdrawal) with negative thyroglobulin antibodies [8]. Thyroglobulin measurements were performed using a chemiluminescence immunoassay (Immulite 2000, Diagnostics Products Corporation). Because an earlier chemiluminescence immunoassay assay used at HVMA had a lower limit of detection of 0.9 μg/L, an unstimulated thyroglobulin level of 0.9 μg/L or greater was used to define tumor recurrence in individuals whose most recent serum thyroglobulin was obtained prior to the assay change. Imaging modalities also used to define recurrence or persistence were whole body radioiodine scan with any positive uptake, a positive PET scan, and/or a positive neck ultrasound scan with confirmatory fine needle aspiration biopsy or surgical pathology of tissue sampled from the suspected site(s) of recurrence.

### Statistical analysis

This study was a retrospective chart review of a preexisting database, and therefore it was not powered for the primary outcome. Descriptive information is presented as frequencies and percentages for categorical variables and as means ± SD for continuous variables. Age was analyzed as a continuous variable in the primary analysis and dichotomized at 45 years for some of the secondary analyses in the Cox proportional hazard regression models. Tumor size was dichotomized at <1 cm and ≥ 1 cm. Secondary analyses included additional stratifications of tumor size at <1.5 cm and ≥ 1.5 cm, <2 cm and ≥ 2 cm, <3 cm and ≥ 3 cm, <4 cm and ≥ 4 cm; tumor size was also analyzed as a continuous measure. Differences between categorical variables were analyzed using the Chi-square test. Among subjects in whom either the month or date of thyroid surgery was undocumented (but for whom at least the year was recorded), the missing month was assigned as June and the date as the 15th of the month. Study entry was the date of surgery. Follow-up was accrued until the first occurrence of the primary outcome or the date of last contact.

Independent predictors were organized into two conceptual groups (patient-associated and tumor-associated predictors). Separately and together in a comprehensive model, Cox proportional hazards regression models and the appropriate tests for verifying the proportional hazards assumption were constructed to determine the associations of the independent predictors with the time to tumor persistence or recurrence. Person-time was computed to the first recurrence and was censored at the date of last contact. Models were adjusted for sex, race, and subjects' age at surgery. Potential confounders were assessed for as appropriate. A subset analysis of the two conceptual groups, again separately and together in a comprehensive model, was also performed excluding patients who had thyroid surgery prior to the start of the electronic database to minimize any selection bias due to subjects who may have died (from WDTC or otherwise) prior to the start of the study period.

P-values <0.05 were considered statistically significant. All statistical analyses were performed using SAS version 9.1 (SAS Institute, Cary, NC).

## Results

From the database of 1,025 patients, 431 met entry criteria. The 594 excluded patients were comprised of 220 patients who did not have a total or near-total thyroidectomy or postoperative radioiodine ablation (groups in whom Tg levels are difficult to interpret), 152 patients who had no laboratory results or imaging studies for thyroid cancer at least 6 months after thyroid surgery internally at HVMA, 68 patients who had benign thyroid disease or a non-thyroidal cancer, 52 patients who had no surgical pathology results in the HVMA database, 46 patients without a recorded year of surgery, 27 patients who were under age 21 at the time of surgery, 15 patients who had medullary thyroid carcinoma, 5 patients who had anaplastic thyroid carcinoma, 8 patients who had positive Tg-antibody titers without follow-up imaging, and 2 patients who were miscoded with an incorrect medical record number. Descriptive statistics of the study population are given in Table 1.

**Table 1:** Subject demographics, tumor characteristics, and management factors

	n
***Gender***	
Male	110 (26%)
Female	321 (74%)

***Race***	
Caucasian	280 (79%)
Black	34 (10%)
Hispanic	7 (2%)
Asian/Pacific Islander	12 (3%)
Other	21 (6%)

***Age (years) at surgery***	45.8 (mean) ± 13.5 (SD)

***Tumor size (cm)***	2.5 (mean) ± 1.6 (SD) (2.2 (median); range, 0.1-10.0

***Tumor size***	
<1 cm	39 (11%)
≥1 cm	329 (89%)

***Tumor histology***	
Papillary (PTC)	388 (91%)
Follicular (FTC)	20 (5%)
Both PTC and FTC	6 (1%)
Hurthle cell carcinoma (HCC)	9 (2%)
Both PTC and HCC	4 (1%)

***Number of tumor foci***	
1	213 (57%)
≥2	161 (43%)

***Thyroid cancer recurrence or persistence***	
Yes	52 (12%)
No	379 (88%)

***Positive disease on follow-up imaging***	
Yes	33 (9%)
No	334 (91%)

***Years after surgery at recurrence***	6.1 (mean) ± 5.6 (SD) 4.3 (median) 0.2-23.2 (range)

* Total n for each descriptor may not equal 431 due to missing data, and total percentages may not equal 100 due to rounding.

### Survival analysis

**Figure 1:**
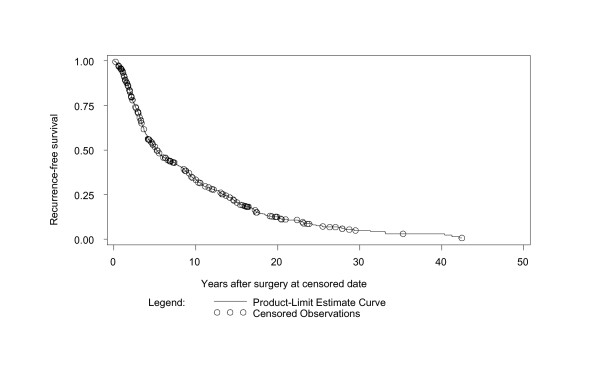
**Kaplan-Meier curve of differentiated thyroid cancer persistence or recurrence (full dataset)**.

There were 2,600 total person-years of follow-up; 52 subjects (12%) had WDTC persistence/recurrence (Figure 1) at a median of 4.3 years (range 0.2-26.2; mean 6.1 ± 5.6 years) after thyroid surgery.

### Multivariate analyses of the full dataset

The Cox regression model of patient-associated predictors of disease persistence or recurrence was significant (p = 0.02), while that of tumor-associated predictors was not (p = 0.80). In a multivariable analysis (with sex, race, age at diagnosis, number of tumor foci, tumor size, and tumor histology as independent predictors), the comprehensive Cox regression model was predictive (p = 0.04) of disease persistence or recurrence, including older age as a significant independent predictor (p = 0.02). The comprehensive models predicting disease persistence or recurrence were also significant when tumor size was categorized into 4 groups (<1 cm, 1 to <2 cm, 2 to <3 cm, and ≥3 cm) (p = 0.04); dichotomized at 1.5 cm (p = 0.04), 2 cm (p = 0.04), 3 cm (p = 0.04), or 4 cm (p = 0.04); and when analyzed as a continuous measure (p = 0.04). Greater age as a continuous variable was weakly predictive of tumor persistence or recurrence, although this was not consistent across all models. A summary of the Cox regression models is presented in Table 2.

**Table 2:** Individual and comprehensive Cox regression models predicting the persistence or recurrence of WDTC

Table 2					
	**n**	**number with persistence or recurrence**	**hazard ratio**	**95% CI**	**p**

***Patient-associated factors***	354	44			0.02
Gender			0.54	0.28, 1.03	0.06
Age			0.97	0.93, 1.01	0.09
Ethnicity			1.17	0.96, 1.42	0.12
Interaction of age and time to persistence or recurrence			1.03	1.01, 1.06	0.01

***Tumor-associated factors***	368	39			0.80
Size (dichotomized)			0.81	0.28, 2.32	0.70
Number of foci			0.88	0.61, 1.27	0.48
Histology			0.89	0.53, 1.49	0.66

***Comprehensive model***	309	37			0.04
Gender			0.57	0.27, 1.21	0.14
Age			0.95	0.90, 0.99	0.02
Ethnicity			1.17	0.96, 1.44	0.13
Tumor size			0.57	0.19, 1.68	0.31
Number of foci			0.94	0.65, 1.36	0.74
Histology			0.68	0.32, 1.44	0.31
Interaction of age and time to recurrence or persistence			1.04	1.02, 1.07	<0.01

### Multivariate analyses after excluding subjects with thyroid surgery prior to 1979

A subset analysis was also performed to include only patients who had surgery after the start of the study period (January 1979) (n = 415). The Cox regression model of patient-associated factors was predictive (p = 0.03), and male gender was an independent positive significant predictor (HR 0.49; 95% CI, 0.25 to 0.95; p = 0.03). The regression model of tumor-associated factors was not predictive (p = 0.85). Similarly, using this restricted cohort, the multivariable analysis (with sex, race, age at diagnosis, number of tumors, tumor size, and tumor histology as independent predictors) were not predictive of disease persistence or recurrence (p = 0.06).

## Discussion

This retrospective analysis suggests that specific factors may predict disease persistence or recurrence in patients with well-differentiated thyroid carcinoma. The American Thyroid Association has recommended that follow-up treatment varies according to a patient's risk for recurrence [8], yet management, including the extent of surgery, necessity of post-operative ablative radioiodine, and degree of thyroid hormone suppression, has been controversial and practices are not uniform among clinicians.

An analysis of all patients with a history of WDTC in our database, regardless of the extent of surgery or use of radioiodine (n = 651), resulted in a persistence/recurrence rate of 20% (data not shown). While this is virtually identical to what was observed in a similar cohort assessed over 30 years by Mazzaferri and Jhiang (21%) [9], by restricting our analyses to patients who underwent a total or near-total thyroidectomy and radioiodine therapy, the rate dropped to 12%. Our Cox regression model of patient-associated factors and the global model were predictive of well-differentiated thyroid cancer persistence or recurrence, although the regression model of tumor-associated factors was not. Greater age as a continuous variable was a weakly predictive factor, but this was not consistent across all models.

Our analysis included only those who were alive at follow-up, and therefore loss of follow-up due to death [both disease-specific and all-cause mortality) was not ascertained. In addition, some subjects excluded due to the absence of thyroglobulin values or an imaging study within the HVMA database were managed by non-HVMA providers and were not available for this analysis. Finally, the unique demographics and socioeconomic status of this study population (e.g. predominantly Caucasian) may limit generalizability to other populations managed by clinicians experienced in thyroid cancer follow-up.

It is difficult to compare the relative predictive abilities of different models or staging systems, as the components in each are not uniform. Palme et al. reported that male sex, advanced initial stage, and presence of extrathyroidal spread were independent predictors of multiple recurrences of well-differentiated thyroid cancer [11]. In a recent retrospective review of papillary microcarcinoma (≤1 cm), Mercante et al. found that capsular invasion, extrathyroidal tumor extension, and neck lymph node metastasis at presentation were the only independent risk factors for the persistence or recurrence of disease [12]. In contrast to these and other studies [13,14], many of the subjects in our study received their surgery at a non-HVMA site, and thus, information regarding extrathyroidal spread was not codified in a uniform manner and could not be analyzed in our cohort. The inability to capture this information may have introduced a misclassification bias of patients' disease burden, thereby altering our findings. The use of a <1 cm cutoff for tumor size in our study (as compared to ≤1 cm) may also limit direct comparison of our results to some studies. Finally, although we did not study these specific questions, it was recently reported that recurrence of papillary and follicular thyroid cancer in the first year following thyroid surgery predicts a worse outcome [13], and patients with micropapillary multifocal thyroid cancer have a reduced rate of recurrence following more complete thyroid surgery [15]. The differences between the variables analyzed in this study and those of other investigators do not allow for direct comparison of the predictive models.

There are several strengths of this study, which specifically studied only patients who underwent a total or near-total thyroidectomy and radioiodine ablation to evaluate laboratory and imaging markers of WDTC persistence/recurrence. All sites of a large Boston, Massachusetts-area multispecialty group practice were included, and there was a relatively long period of follow-up of over 28 years. Many subjects contributed substantial person-time toward the primary outcome. Furthermore, this group practice, which consists of 14 sites, has utilized an electronic medical record since the late 1970s, thus permitting an easily extractable and long-term comprehensive assessment of patients followed within it with WDTC.

The findings of this study do not confirm previously published data that male gender and larger tumors have a worse prognosis for WDTC persistence/recurrence. We urge the establishment of a comprehensive national registry, created through linkage with electronic health record systems, that would uniformly capture information regarding these and other factors for the long-term monitoring of WDTC outcomes.

## List of Abbreviations

CT: is computed tomography; HVMA: is Harvard Vanguard Medical Associates; MRI: is magnetic resonance imaging; FDG-PET: is positron emission tomography; SD: is standard deviation; rhTSH: is recombinant human thyroid stimulating hormone; Tg: is thyroglobulin; WDTC: is well-differentiated thyroid cancer.

## Competing interests

The previously-created health quality assurance HVMA database was funded in part by Genzyme Corporation. Dr. Jeffrey R. Garber has received fees from Abbott Laboratories and King Pharmaceuticals. Ms. Shalini Dave; Drs. Angela M. Leung, Stephanie L. Lee, Francis X. Campion, and Elizabeth N. Pearce have no conflicts of interest to disclose.

## Authors' contributions

AL performed the data analysis and wrote the initial draft of the manuscript. SD obtained approval from the Institutional Review Board and performed the data extraction in preparation for our analysis. SL, FC, and JG helped to establish the creation of the thyroid cancer registry. EP assisted SD in obtaining Institutional Review Board approval and assisted AL in the data analysis and preparation of the manuscript. All authors read and approved the final manuscript.
